# A Novel Gain‐of‐Function GLUL Variant Is Associated With Developmental and Epileptic Encephalopathy With Enlarged Perivascular Spaces

**DOI:** 10.1155/humu/2799390

**Published:** 2026-06-16

**Authors:** Tenghui Wu, Fang He, Xiaoyuan Ni, Fei Yin, Jing Peng

**Affiliations:** ^1^ Department of Pediatrics, Xiangya Hospital, Central South University, Changsha, Hunan Province, China, csu.edu.cn; ^2^ Clinical Research Center for Children Neurodevelopmental Disabilities of Hunan Province, Xiangya Hospital, Central South University, Changsha, Hunan Province, China, csu.edu.cn

**Keywords:** developmental and epileptic encephalopathy, GLUL, glutamine synthetase, perivascular spaces

## Abstract

Two clinical phenotypes are associated with GLUL mutations, from different inheritance mode. Recessive forms are associated with congenital glutamine deficiency, manifesting with severe brain malformation, multiorgan failure, and early death. A dominant form has recently been described, which involves dysregulated glutamine synthetase stability and manifests as developmental and epileptic encephalopathy (DEE). All reported dominant mutations are within the start codon or the 5 ^′^ UTR. Here, we report a DEE patient with a de novo variant, c.522_536dup, in the catalytic domain of GLUL. Her brain MRI demonstrated involvement of the white matter signal‐intensity alterations and markedly enlarged perivascular spaces. In vitro overexpression assays revealed no difference in protein expression or enzyme activity between the mutant and wild‐type under normal glutamine conditions. However, under either low or high glutamine concentrations, the mutant exhibited significantly higher enzyme activity than the wild‐type, indicating disrupted regulation of glutamine synthetase activity. This study expands the spectrum of variants, provides further evidence for the role of enzyme stability in gain‐of‐function variants, and highlights enlarged perivascular spaces as a diagnostic clue in GLUL‐related disorders.

## 1. Introduction

Glutamine synthetase (GS), encoded by GLUL (OMIM: 138290), is highly expressed in the glial cell types of the central nervous system. Under physiological circumstances, homeostasis of glutamine and glutamate in the brain is strictly regulated. Glutamate is excreted by neurons into the synaptic cleft as a neurotransmitter and absorbed by astrocytes, where it is converted into glutamine by GS. Glutamine is then transported toward neurons and again converted into glutamate by glutaminase to restart signal transduction. Disturbed glutamate homeostasis is detrimental for neurological functioning and leads to a severe neurological phenotype.

Glutamine deficiency (OMIM #610015), caused by pathogenic variants in the GLUL gene, is a rare autosomal recessive (AR) inborn error of metabolism characterized by glutamine deficiency and hyperammonemia [1]. Since 2005, eight cases of glutamine deficiency have been reported thus far [1–5]. Most individuals with biallelic GLUL variants presented with neonatal‐onset symptoms, including seizures, global developmental delay, and even multiorgan failure and early lethality. Brain MRI findings include brain malformations, ventriculomegaly, and may be accompanied by cystic lesions. The clinical severity seems correlate with markedly reduced glutamine levels in plasma and cerebrospinal fluid. A homozygous GLUL gene deletion was reported prenatally, associated with fetal growth restriction, severe brain malformations, and multisystem congenital anomalies [2].

In 2024, de novo variants in GLUL were first found in a patient with developmental and epileptic encephalopathy (DEE) [6]. All reported de novo variants were located in the start codon or the 5 ^′^ UTR, affecting the stabilization of GS. This nonlethal outcome, together with the absence of major structural brain anomalies, suggests that these patients may retain higher residual GS enzyme activity. Indeed, in contrast to the loss‐of‐function (LoF) mechanism observed in recessive cases, these autosomal dominant (AD) variants exhibit a gain‐of‐function (GoF) effect. These de novo variants result in the escape of GS from glutamine‐mediated negative feedback and ubiquitin‐proteasomal degradation, leading to its aberrant stabilization and activity [6].

Here, we report the first patient with a pathogenic de novo variant in the catalytic domain of GLUL. The clinical presentation is characterized by DEE, accompanied by markedly enlarged perivascular spaces (EPVS).

## 2. Materials and Methods

### 2.1. Informed Consent

This research was approved by the ethics committee of Xiangya Hospital of Central South University (202307148) and has been performed following the ethical standards defined in the Declaration of Helsinki. Informed consent was obtained from the parents.

### 2.2. Clinical and Genetic Investigations

The available medical records and MRI were reviewed. Genomic DNA extracted from blood of the patient and parents, was sequenced on Illumina NovaSeq platform with at least Q20 base quality, and > 30 × mean coverage. Sequences were compared to the human reference genome GRCh38/HG38 for true and false variants identification. BAM files were locally recompared using GATK series software. Annovar was used to annotate VCF variation files. Principles for screening pathogenic mutation: (1) to screen out exon region mutation and nonsynonymous mutation; (2) to screen out variants not observed or less than 5% in ExAC_EAS, ExAC_ALL, 1000Genomes, and gnomAD databases; (3) to evaluate variants according to dbSNP, OMIM, HGMD, ClinVar, and other databases; (4) to predict the protein function by SIFT, Polyphen2, MutationTaster, and other protein function prediction software. Pathogenic variants were screened according to ACMG classification guidelines and patients′ clinical phenotypes [7]. The identified variants were subsequently confirmed by Sanger sequencing.

### 2.3. Structural Comparison

The wild‐type (WT) sequence was retrieved from UniProt. The in‐frame duplication was then introduced into this sequence to generate the mutant sequence. The mutant sequence was used as input for AlphaFold3 to predict its three‐dimensional structure [8]. The predicted mutant structure was aligned with the WT human GS bound to ADP at 2.60 Å resolution (PDB entry 2QC8). Illustrations were prepared with PyMOL (Version 3.1.1 Schrödinger LLC). The binding energies of WT and mutant GLUL with glutamic acid were calculated using the DockingPie 1.2 [9] plugin within PyMOL. *ΔΔ*G scores for the in‐frame duplication were calculated using FoldX [10].

### 2.4. Cell Culture and Transfection

HEK293 cells were maintained in DMEM media (Servicebio, #G4515) supplemented with 2% penicillin/Streptomycin (Gibco, #15140122), 10% fetal bovine serum (FBS) (Procell, #164250), and 4 mM glutamine (Servicebio, #GM3031) at 37 °C in 5% CO_2_. Human GS (GenBank: NM_001033044.4) expression constructs with WT, c.522_536dup, or c.1021C > T were cloned into pcDNA3.1 vectors with 3 × FLAG. HEK293 cells were transfected with these constructs using Lipofectamine 2000 (ThermoFisher, #11668019) according to the manufacturer′s instructions. To evaluate the regulation of GS enzyme activity across different glutamine concentrations, after transfection, the medium was replaced with DMEM media (Servicebio, #G4517) supplemented with 2% penicillin/streptomycin (Gibco, #15140122), 10% FBS (Procell, #164250), and varying concentrations of L‐glutamine (1, 2, 4, 16, 32, and 64 mM) (Servicebio, #GM3031). Cells were harvested 24 h posttransfection for GS activity assays.

### 2.5. Western Blotting

Cells were lysed in RIPA buffer (Beyotime Biotechnology, China) containing 1 mM phenylmethanesulfonyl fluoride (Beyotime Biotechnology, China)) on ice for 30 min. Ten micrograms of protein lysate were resolved in 10% SDS‐polyacrylamide gel and transferred to polyvinylidene fluoride membrane. The membrane was probed with primary antibody (Anti‐Flag, Sigma, #SAB4301135; Anti‐GAPDH, Proteintech, #HRP‐60004) at 4°C overnight after incubation for 10 min in protein‐free blocking buffer (Epizyme, #PS108P) at room temperature. The secondary antibody was applied for 1 h following the removal of the primary antibody, and finally, the signals were revealed by chemiluminescent detection.

### 2.6. GS Activity Assay

Following transfection, samples were measured for GS activity using the GS activity assay kit (Solarbio, #BC0910) as per the manufacturer instructions.

### 2.7. Statistical Analysis

Statistical analyses were performed using GraphPad Prism 8. Comparisons between multiple groups were analyzed by two‐way ANOVA followed by Dunnett′s multiple comparisons test, with WT as the control group at each glutamine concentration. Data are presented as mean ± SEM from at least three independent biological replicates. *p* < 0.05 was considered statistically significant.

## 3. Results

### 3.1. Clinical Presentation

An eight‐month‐old girl was referred to our hospital with focal seizures and developmental delay. She was initially treated with topiramate, but her seizures were poorly controlled and she experienced an episode of status epilepticus. Clobazam was then added, which kept her seizure‐free for 1 year.

She was the first child of nonconsanguineous and healthy parents. Both parents were clinically examined and reported no history of seizures, developmental delays, or other neurological symptoms. She had a normal prenatal follow‐up and was born at term via an uneventful delivery with a birth weight of 3000 g. At 1 year old, she weighed 10 kg, and was 77 cm tall with a head circumference of 44 cm, but had not yet achieved the milestones of sitting or speaking. She had no dysmorphic features, and neurologic examination revealed hypotonia. Vision and hearing assessment were normal. At the last examination at 2 years old, she had shown no developmental progress and still could not sit.

**Figure 1:**
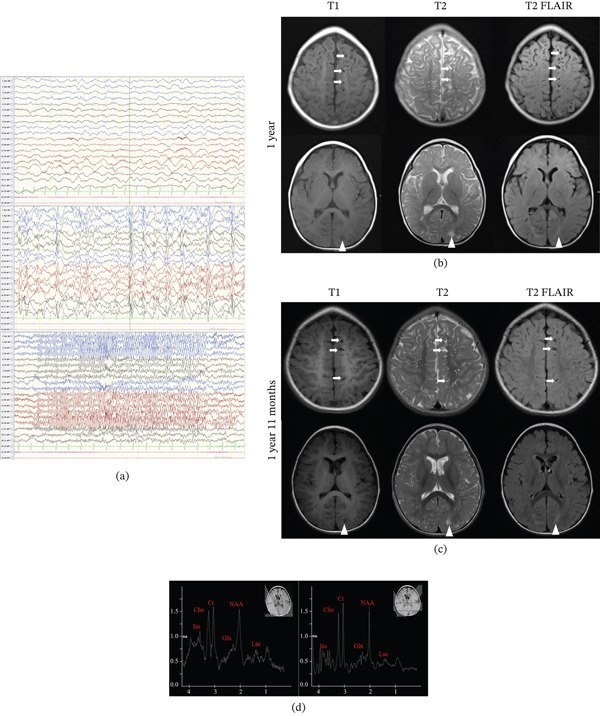
EEG and MRI features. (a) EEG shows slow background activity along with diffuse *δ* activity with multifocal epileptiform activity. Serial neuroimaging (b and c) at Ages 1 year, and 1 year 11 months, including axial T1, T2, and fluid attenuated inversion recovery T2 (Column 3). Extensive EPVS are seen in frontal and parietal lobes (white arrow), without significant progression over time. White matter hyperintensity (white arrowheads) are seen in the bilateral cerebral ventricle occipital horns: T1 hypointensity, T2 hyperintensity, and FLAIR T2 hyperintensity. (d) MR spectrum obtained from the right basal ganglia and left white matter hyperintensity shows no significant difference. Resonance signals from mobile lipids (Lip; 0,9 and 1,3 ppm), lactate (Lac; 1,3 ppm), alanine (Ala; 1,5 ppm), N‐acetylaspartate (NAA; 2,0 ppm), complex glutamates/glutamine (Glx; 2.02–2.5 and 3.65–3.8 ppm), creatine (Cr; 3.0 ppm), choline‐containing compounds (Cho; 3.2 ppm) and myoinositol (mI; 3.56 ppm) were obtained.

Electroencephalogram (EEG) showed slow background activity along with diffuse *δ* activity with multifocal epileptiform activity (Figure 1a). Brain MRI showed paraventricular white matter hyperintensities and multiple EPVS in frontal and parietal lobes (Figures 1b,c). MRS was performed at 1 year 11 months, and the voxel was placed centrally within the white matter hyperintensities, avoiding the cystic component. The peaks height was evaluated qualitatively in comparison with Cr peak when detected, however, no significant difference is found (Figure 1d).

Metabolic investigations including blood amino acids, blood acylcarnitine profiles, and urinary organic acid analysis were performed using mass spectrometry. Blood glutamine level was 14.59 (1.00–55.00) and glutamate level was 59.53 (45.00–280.00). Plasma ammonia level was 37.1 *μ*mol/L (18–72 *μ*mol/L). Very long chain fatty acids and evaluation for lysosomal storage diseases were performed. All results were within reference ranges except for a low plasma lysosomal acid alpha‐glucosidase of 10.05 nmol/1 h/mg (> 14 nmol/1 h/mg).

**Figure 2:**
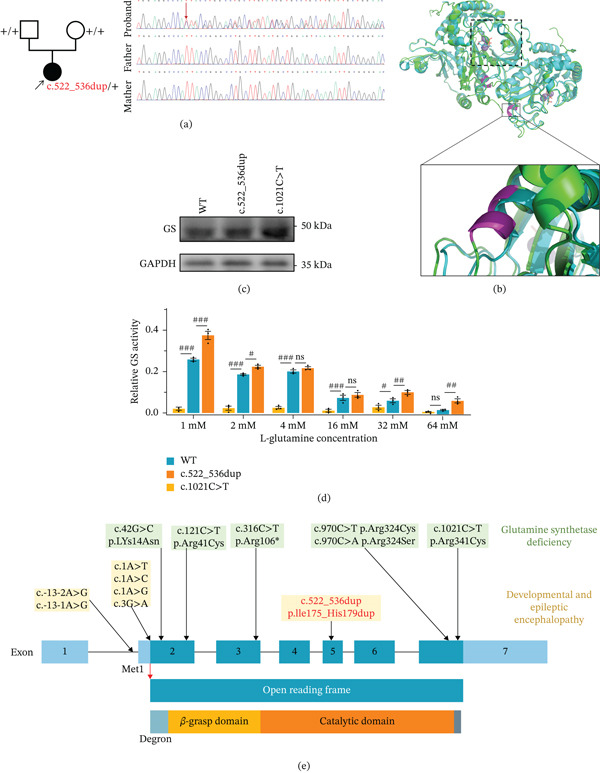
Genetic and structural characterization of the GLUL c.522_536dup variant. (a) Pedigree and Sanger sequencing chromatograms. The sequence traces from the proband (upper panel) show a double peak starting from the duplication site, consistent with heterozygosity. (b) Superimposition of the mutant GS dimer (green) onto the WT GS structure (PDB 2QC8, blue). Black dotted lines indicate the active site. The magnified view on the right shows the mutated region, with the five duplicated amino acids (p.Ile175_His179dup) highlighted in magenta. (c) Western blot analysis of GS protein expression in HEK293 cells transfected with WT, c.1021C > T, or c.522_536dup constructs. GAPDH was used as a loading control. (d) GS enzyme activity in HEK293 cells transfected with WT, c.1021C > T, or c.522_536dup constructs, measured across a range of L‐glutamine concentrations (1, 2, 4, 16, 32, and 64 mM). The c.1021C > T variant showed markedly reduced activity at all concentrations except 64 mM. The c.522_536dup variant exhibited significantly higher activity than WT at 1 mM (*p* < 0.0001), 2 mM (*p* < 0.05), 32 mM (*p* < 0.01), and 64 mM (*p* < 0.01), with no significant difference at 4 or 16 mM. ^#^ *p* < 0.05; ^##^ *p* < 0.01; ^###^ *p* < 0.0001; ns, not statistically significant. (e) Location of the GLUL variants. The variant c.522_536dup identified in this study is indicated in red. Seven variants previously reported in AR GS deficiency are shown against a green background, and six variants associated with AD DEE are shown against a yellow background.

In 2023, trio‐based whole exome sequencing did not identify a diagnostic variant. At the second year, data reanalysis revealed that the patient had a heterozygous de novo variant (c.522_536dup, p.Ile175_His179dup) of the GLUL gene, following description of a dominant disorder for GLUL by Jones et al. This variant was subsequently confirmed by Sanger sequencing (Figure 2a).

### 3.2. Structural Comparison

In the crystal structure, GS forms a pentamer, which is associated with another pentamer in a head‐to‐head configuration to form a decamer. The interface forming the active site is built by a *β*‐*β* interaction between the N‐terminal *β*‐grasp domain of one subunit and the highly curved *β*‐sheet of the C‐terminal catalytic domain in the adjacent subunit so as to form a funnel‐shaped pocket [11]. The crystal structure of mutant GS subunits displayed high similarity to the subunits of GS (PDB entry 2QC8) (Figure 2 b), with RMSD of 0.336 Å. The mutant exhibited a binding energy of −5.397 kcal/mol for glutamic acid, which is nearly identical to that of the WT (−5.382 kcal/mol). In combination with FoldX, the AlphaFold3 model yields the prediction that the p.Ile175_His179dup mutation enhances protein stability, with the mutant exhibiting a *ΔΔ*G value of −8.15 kcal/mol relative to WT GLUL.

### 3.3. Enzyme Activity

Given that de novo monoallelic variants in the 5 ^′^ region of GLUL cause a severe neurodevelopmental disorder, whereas heterozygotes for LoF alleles are unaffected, we hypothesized that the c.522_536dup variant confers a GoF mechanism. We evaluated this by comparing it to the WT and the c.1021C > T variant, a known LoF control from patients with AR GS deficiency.

Both the c.522_536dup and c.1021C > T variants produced protein levels comparable to WT (Figure 2c). To further characterize the functional impact of these variants, we expressed WT, c.1021C > T, or c.522_536dup in HEK293 cells and measured GS enzyme activity across a range of L‐glutamine concentrations (1, 2, 4, 16, 32, and 64 mM) (Figure 2d). As expected, WT GS activity showed a progressive decline with increasing glutamine concentrations. The c.1021C > T variant showed markedly reduced activity at all concentrations except 64 mM, where WT activity was already suppressed at high glutamine concentration. In contrast, the c.522_536dup variant exhibited significantly higher activity than WT at 1 mM, 2 mM, 32 mM, and 64 mM, with the most pronounced difference at 1 mM. The c.522_536dup variant failed to exhibit this concentration‐dependent downregulation, maintaining elevated activity at both low and high concentrations. Overall, these data suggest that the c.522_536dup variant causes a dysregulation of GS feedback by glutamine, leading to inappropriately high enzyme activity across a broad range of extracellular concentrations.

### 3.4. Phenotypic Comparison

The locations of all reported variants are shown in Figure 2e. Table 1 provides a comparison of the phenotype in AR and AD patients. Regarding the age of onset, AR patients typically present in the neonatal period or early infancy (birth to 5 months), whereas AD patients have a broader range of onset ages, mostly concentrated in mid‐to‐late infancy (2–11 months). DEE and global developmental delay are common features under both inheritance patterns. However, the reported seizure types are more diverse in AD cases. Notably, brain MRI features show high consistency in AD cases, consistently characterized by prominent perivascular spaces accompanied by myelination abnormalities and thinning of the corpus callosum. In contrast, the MRI presentations in AR cases are more heterogeneous and severe, including markedly immature brain development, atrophy, multiple periventricular cavitations, and severe cerebellar hypoplasia. Furthermore, severely low serum glutamine levels and systemic features such as cardiac insufficiency and epidermal necrolysis are almost exclusively observed in AR cases. In summary, the AD form of the disease predominantly manifests as a relatively confined phenotype centered on the nervous system, whereas the AR form often predicts a more severe clinical course involving multiple systems.

**Table 1:** Clinical and biochemical characteristics of the nineteen known cases of GLUL mutation.

Genetic change	Number of patient	Inheritance pattern	Variant origin	Consanguinity	Sex	Age at presentation	DEE	Seizure type	Global developmental delay	Hypotonia	Other features	Serum glutamine	Brain MRI	Age at last assessment	Ref.
c.970C > T, p.Arg324Cys; hom	2	AR	Inherited	Yes	2 Male	Birth	Yes	Neonatal seizures	Yes	Yes	Cardiac insufficiency, dysmorphic features, musculoskeletal abnormality	2 *μ*M (433–619 *μ*M)	A markedly immature brain with hyperintensity of the white matter, enlarged lateral ventricles, paraventricular cysts, a small, smooth cerebellum, and large pericerebral spaces.	2 days	[1]
c.1021C > T, p.Arg341Cys; hom	1	AR	Inherited	Yes	Female	Day 1	Yes	Neonatal seizures	Yes	Yes	Dysmorphic features, respiratory failure, epidermal necrolysis	6 *μ*M (300–800 *μ*M)	Markedly attenuated gyri and subependymal cysts.	28 days	[1]
c.970C > A, p.Arg324Ser; hom	1	AR	Inherited	Yes	Male	Day 1	Yes	GTCS	Yes	Yes	—	126 *μ*M (376–709 *μ*M)	Hypomyelination of the white matter and mild degree of brain atrophy with prominent cortical sulci and sylvian fissures.	6 years	[1]
c.121C > T, p.Arg41Cys; hom	1	AR	Inherited	Yes	Female	5 months	Yes	Epileptic spasm	Yes	Yes	Necrolytic erythema	297 *μ*M (300–800 *μ*M)	Periventricular hyperintensities adjacent to the posterior horn (35 months old).	3 years	[3]
Del 1q25.3; hom	1	AR	Inherited	Yes	Female	15 + 5 weeks gestation	—	—	Yes	—	Dysmorphic features, musculoskeletal abnormality	92 *μ*M (451–1113 *μ*M)	Callosal hypoplasia, a thin brainstem with pontine hypoplasia, vermian hypoplasia, multiple periventricular cavitations, severe cerebellar hypoplasia, and markedly simplified gyral pattern (27 weeks′ gestation)	Pregnancy terminated at 29 weeks	[2]
c.316C > T, p.Arg106∗; c.42G > C, p.Lys14Asn	2	AR	Inherited	No	Female	9 months	Unknown	Myoclonic	Yes	Normal	—	Unknown	Normal	12 years and 21 years	[4]
c.1A > T, p.Met1?	1	AD	De novo	No	Female	8 months	Yes	Focal, generalized	Yes	Yes	—	Borderline low	Prominent perivascular spaces and thinning corpus callosum, demyelination/hypomyelination.	Age range 16 months–25 years	[6]
c.1A > C, p.Met1?	1	AD	De novo	No	Female	Unknown	Unknown	Unknown	Yes	Yes	—	Unknown	Unknown		[6]
c.1A > G, p.Met1?	4	AD	De novo	No	Female	10 weeks ~ 8 months	Yes	Tonic, focal clonic, myoclonic atonic, GTCS, epileptic spasm	Yes	Yes	—	Normal	Prominent perivascular spaces and thinning corpus callosum, demyelination/hypomyelination.		[6]
c.3G > A, p.Met1?	1	AD	De novo	No	Female	2 months	Yes	GTCS	Yes	Yes	—	Normal	Prominent perivascular spaces and thinning corpus callosum, demyelination/hypomyelination.		[6]
c.‐13‐2A > G	2	AD	De novo	No	1 Female, 1 male	5 ~ 11 months	Yes	Myoclonic, GTCS, absence, tonic‐clonic, tonic	Yes	Yes	Cortical visual impairment, musculoskeletal abnormality	1 normal, 1 unknown	Normal at Ages 1 and 3 years		[6, 12]
c.‐13‐1A > G	1	AD	De novo	No	Male	6 months	Yes	GTCS	Yes	Yes	—	Borderline low	Prominent perivascular spaces and thinning corpus callosum, demyelination/hypomyelination.		[6]
c.522_536dup p.Ile175_His179dup	1	AD	De novo	No	Female	8 months	Yes	Focal clonic	Yes	Yes	—	14.59 *μ*M (1.00–55.00 *μ*M)	Prominent perivascular spaces and paraventricular white matter changes.	2 years	This study

Abbreviations: DEE, developmental and epileptic encephalopathy; hom. homozygous; AR, autosomal recessive; GTCS, generalized tonic‐clonic seizure; AD, autosomal dominant.

## 4. Discussion

In sum, this study further expands the spectrum of dominant GLUL variants associated with DEE. In contrast to AR GS deficiency, the AD form lacks characteristic biochemical abnormalities, such as alterations in plasma ammonia or glutamine levels in the blood and cerebrospinal fluid. Correspondingly, MRS reveals no significant Glx peak abnormalities. Our cellular findings align with these clinical observations, demonstrating that the mutant GS retains enzymatic activity comparable to the WT under normal culture conditions. This suggests a distinct pathological mechanism centered on dysregulation rather than a loss of function. The predicted structural models indicate that the GLUL c.522_536dup variant induces no significant changes in the overall structure of the GS decamer, but that the mutation leads to increased stability of GS, which may contribute to a GoF mechanism. However, we cannot exclude the possibility that additional local structural alterations at other positions may also be responsible for the enhanced function.

A summary of the clinical presentations from the reported cases indicates that EPVS is the most frequent neuroimaging finding, which may be accompanied by delayed myelination and white matter hyperintensities [6]. Particularly in our patient, the MRI revealed prominent honeycomb‐like changes. EPVS appear hypointense on T1‐weighted and T2‐FLAIR images and hyperintense on T2‐weighted images, which are sometimes described as extensive cystic changes. In patients with AR GLUL mutations, although they often exhibit more severe structural malformations such as cerebral dysgenesis, some cases have also been reported to present with paraventricular cysts and cavitations [1, 2]. Therefore, these extensive cystic changes should alert clinicians to this potential diagnosis.

Although EPVS can be a normal finding in healthy individuals, usually measuring < 2 mm in diameter [7], their clinical significance must be interpreted within the appearance of the adjacent tissue on MRI and the clinical context [13]. In GLUL‐related DEE, EPVS accompanied by delayed myelination or white matter hyperintensities form a characteristic feature. EPVS is MRI marker of impaired glymphatic clearance and has been associated with neurodegenerative diseases [14]. The exact mechanisms leading to EPVS have not yet been fully elucidated, and it has been suggested that fluid accumulation in the PVS occurs due to an impairment of perivascular brain clearance [15]. Increasing evidence suggests that soluble waste products are cleared from the brain along the PVS, and that pathological changes in the walls of blood vessels (such as arteriosclerosis, lipohyalinosis, and vascular amyloid *β* (A*β*) accumulation), cause impairment in perivascular clearance [16–18]. Alzheimer′s disease serves as a notable example in which abnormal accumulation of misfolded A*β* is associated with EPVS [19, 20]. In addition, EPVS is also observed in mucopolysaccharidoses, where their dilation is significantly associated with ventriculomegaly, ventricular CSF volume, and elevated aqueductal CSF stroke volume [21].

Perivascular spaces serve as a waste clearance system that is dependent on aquaporin‐4 (AQP4) expressed in astrocytes. In Alzheimer′s disease, perivascular astrocyte endfeet swelling and elevated AQP4 expression have been reported, which may compromise BBB integrity and lead to impaired perivascular clearance [22]. In neuromyelitis optica spectrum disorder, EPVS is found in association with the characteristic AQP4‐mediated astrocyte injury [23].

From a pathophysiological perspective, the GoF mutation in GLUL may lead to excessive glutamine synthesis in astrocytes. Elevated intracellular glutamine acts as an osmolyte, causing astrocytic swelling. This mechanism is observed in hyperammonemic conditions, where increased GS activity drives glutamine accumulation and subsequent astrocyte enlargement [24]. Notably, both GoF and LoF of GLUL have been linked to astrocytic and vascular dysfunction. It has been shown that astrocytic GLUL deletion leads to astrogliosis, along with increased vascular caliber and impaired vasodilation in response to CO_2_ [25]. Genetic deletion of GLUL in endothelial cells in mice impairs vessel sprouting during vascular development [26]. Although the association between GLUL and AQP4 in astrocytes and endothelial cells remains unclear, these findings collectively suggest that dysfunctional glutamate‐ammonia metabolism affects both astrocytes and endothelial cells function and initiates glymphatic impairment and contribute to the formation of EPVS. A limitation of this study is that our functional assays were performed in HEK293 cells rather than in astrocytic models. Transient transfection of GLUL plasmids in C8‐D1A, Neuro‐2a, and SH‐SY5Y cells using different reagents failed due to low efficiency, limiting our ability to assess the variant′s effects in a more physiologically relevant context. Lentiviral transduction or electroporation should be employed in future studies to overcome this limitation and to directly evaluate the pathogenic mechanism of the c.522_536dup variant in the central nervous system.

Methionine sulfoximine, an irreversible GS inhibitor, has therapeutic potential by targeting the GoF caused by GLUL mutation. In hyperammonemia, elevated glutamine synthesized by astrocytic GS, is thought to cause astrocyte swelling and encephalopathy [24], and methionine sulfoximine has successfully been trialed as a therapeutic intervention [27]. A recent study demonstrates that MSO suppresses pilocarpine‐induced seizures by preventing seizure‐associated cell swelling. This finding also suggests the potential of MSO for treating disorders caused by GLUL GoF mutations [28].

## 5. Conclusions

Under physiological circumstances, homeostasis of glutamine and glutamate in the brain is strictly regulated. It is therefore not surprising that disturbed glutamate homeostasis, either by GoF or LoF of GS, is detrimental for neurological functioning. In conclusion, the report of this novel in‐frame duplication provides further supporting evidence for the association of GLUL hyperfunction with impaired PVS, broadening the range of variants associated with GLUL‐DEE and demonstrating the critical role of proper regulation machinery function of GS.

## Author Contributions

T.W. and J.P. designed the research study. T.W. wrote the manuscript. T.W. and X.N. collected clinical and genetic data, and conducted cell experiments. J.P. provided clinical phenotyping and diagnostic data.

## Funding

This study is supported by the National Natural Science Foundation of China, 10.13039/501100001809, 82071462; and China Postdoctoral Science Foundation, 10.13039/501100002858, 2025M782312.

## Disclosure

All authors discussed the results and commented on the manuscript.

## Conflicts of Interest

The authors declare no conflicts of interest.

## Data Availability

The data that support the findings of this study are available from the corresponding author upon reasonable request.
